# Supplementary material for: Barrier-on-a-Chip with a Modular Architecture and Integrated Sensors for Real-Time Measurement of Biological Barrier Function
Source: Micromachines (Basel). 2021 Jul 12;12(7):816. doi: 10.3390/mi12070816 (PMC8305171; doi:10.3390/mi12070816)
Supplement: Supplementary file 1 [file micromachines-12-00816-s001.zip › micromachines-1279706-supplementary.pdf]

micromachines

Supplementary material

Barrier-on-a-Chip with a Modular Architecture and Integrated Sensors for Real-Time Measurement of Biological Barrier Function

Patrícia Zoio, Sara Lopes-Ventura and Abel Oliva

Table S1. Summary of the parameters used in the simulation study.

Name/Description	Symbol	Expression/Value
Medium electrical conductivity	σ_m	1.5(S/m)
Medium relative permittivity	ε_m	78
Tissue height	h	100 (μm)
Normalized transepithelial electrical resistance	TEER.A	10^1 - 10^4 (Ω.cm^2)
Current	I	1 (A)
Tissue conductivity	σ_t	$\frac{h}{TEER.A}$ (S/m)

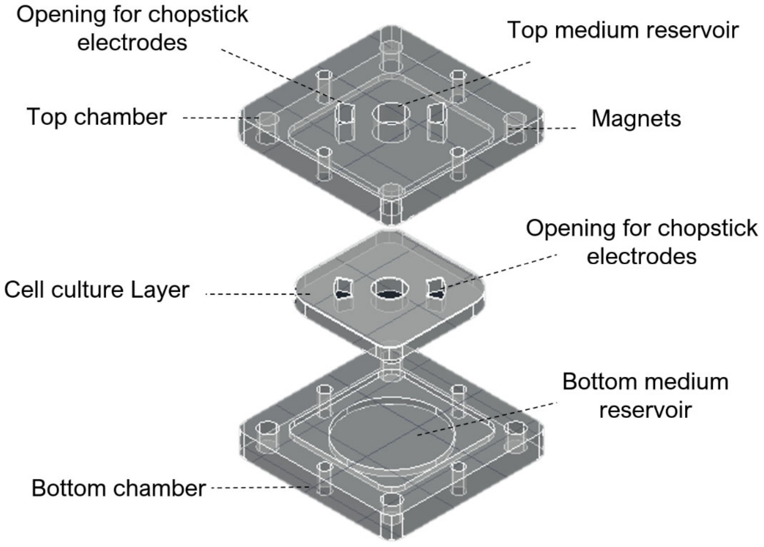

Figure S1. Schematic exploded view of the calibration chamber with openings to insert chopstick electrodes for TEER measurements. The calibration chamber consists of a top and bottom chamber, including a top and bottom medium reservoir and opening for chopstick electrodes. The chamber was designed to accommodate the PDMS insert layer while allowing for the same placement of the chopstick electrodes as in a Transwell®.

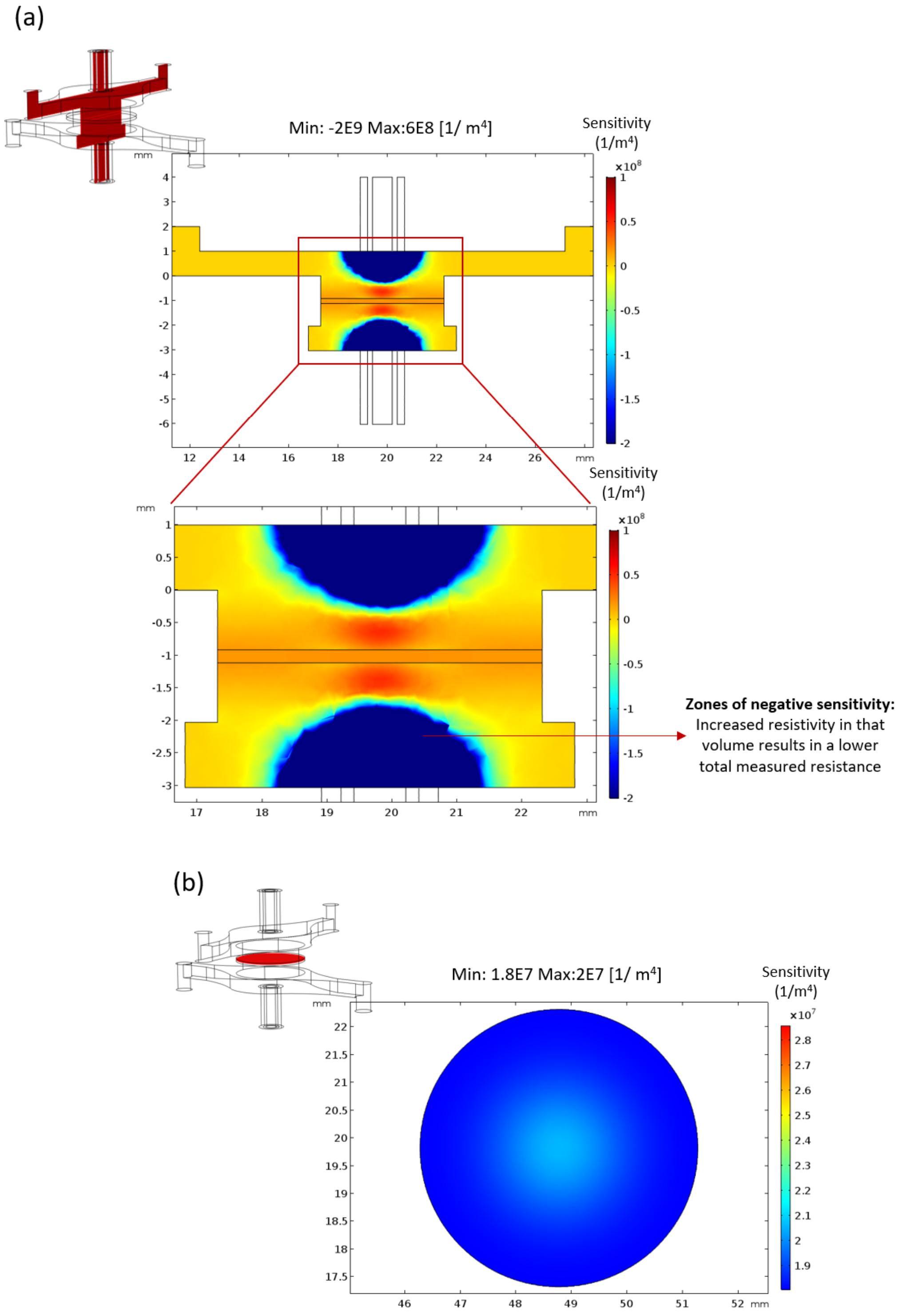

Figure S2. Sensitivity distribution (rainbow colour map) using the BoC A and TEER 10^3 Ω.cm^2 along (**a**) a longitudinal cut in the y–z plane and (**b**) along the cell barrier in the x–y plane.

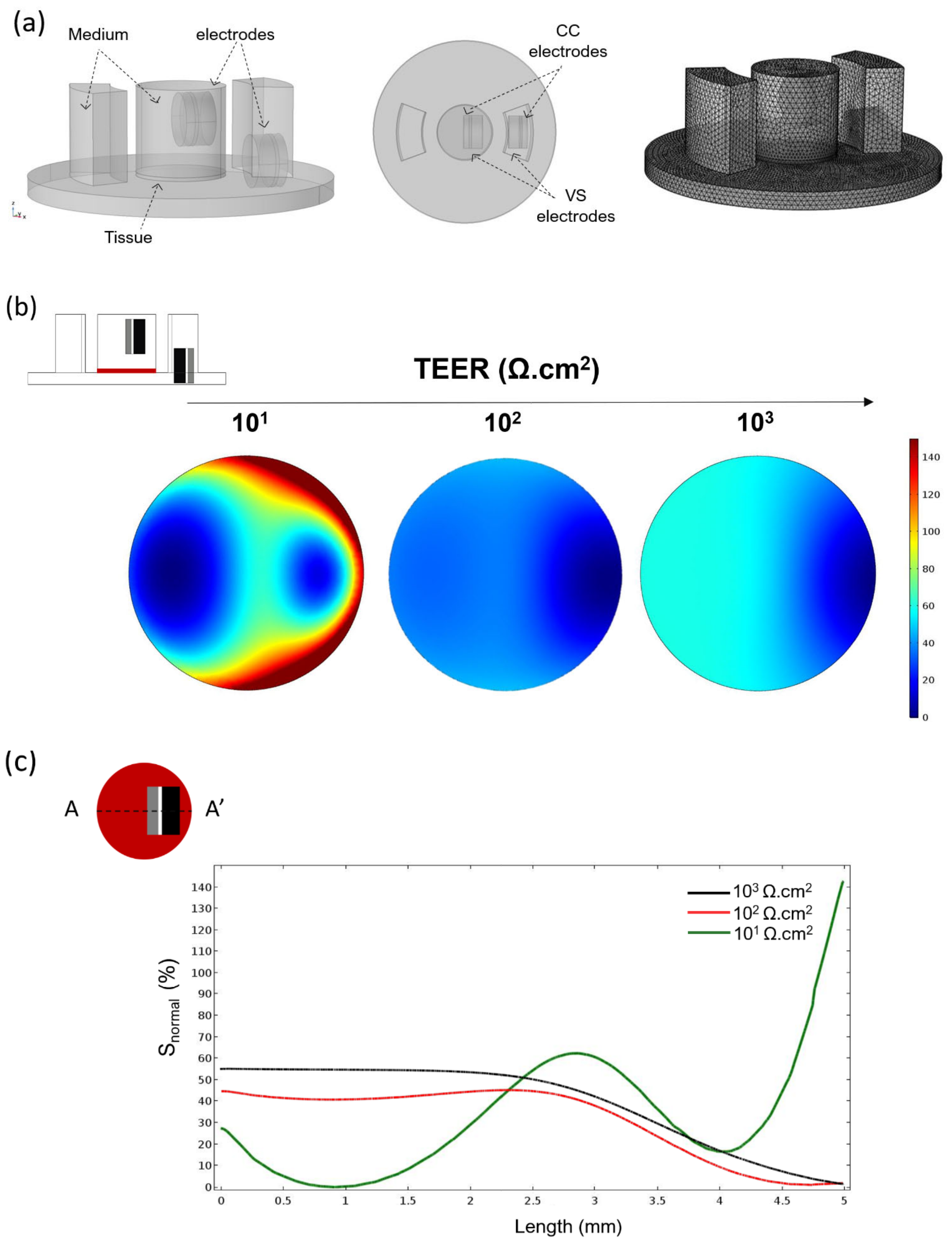

Figure S3. TEER measurement calibration device. (**a**) 3D geometry and created mesh for finite element analysis (FEA). (**b**) Normalized sensitivity distribution (S_{norm}), including the planar sensitivity distribution along the cell barrier surface (rainbow colour map) in the x–y plane and sensitivity distribution along the cell barrier though the axis (lines AA′) shown in the scheme of the model at the left. Results are presented for different TEERS (10^1 -10^3 Ω.cm^2). A S_{norm} value of 0% represents the zone of lower sensitivity. Note that axes have different scales.

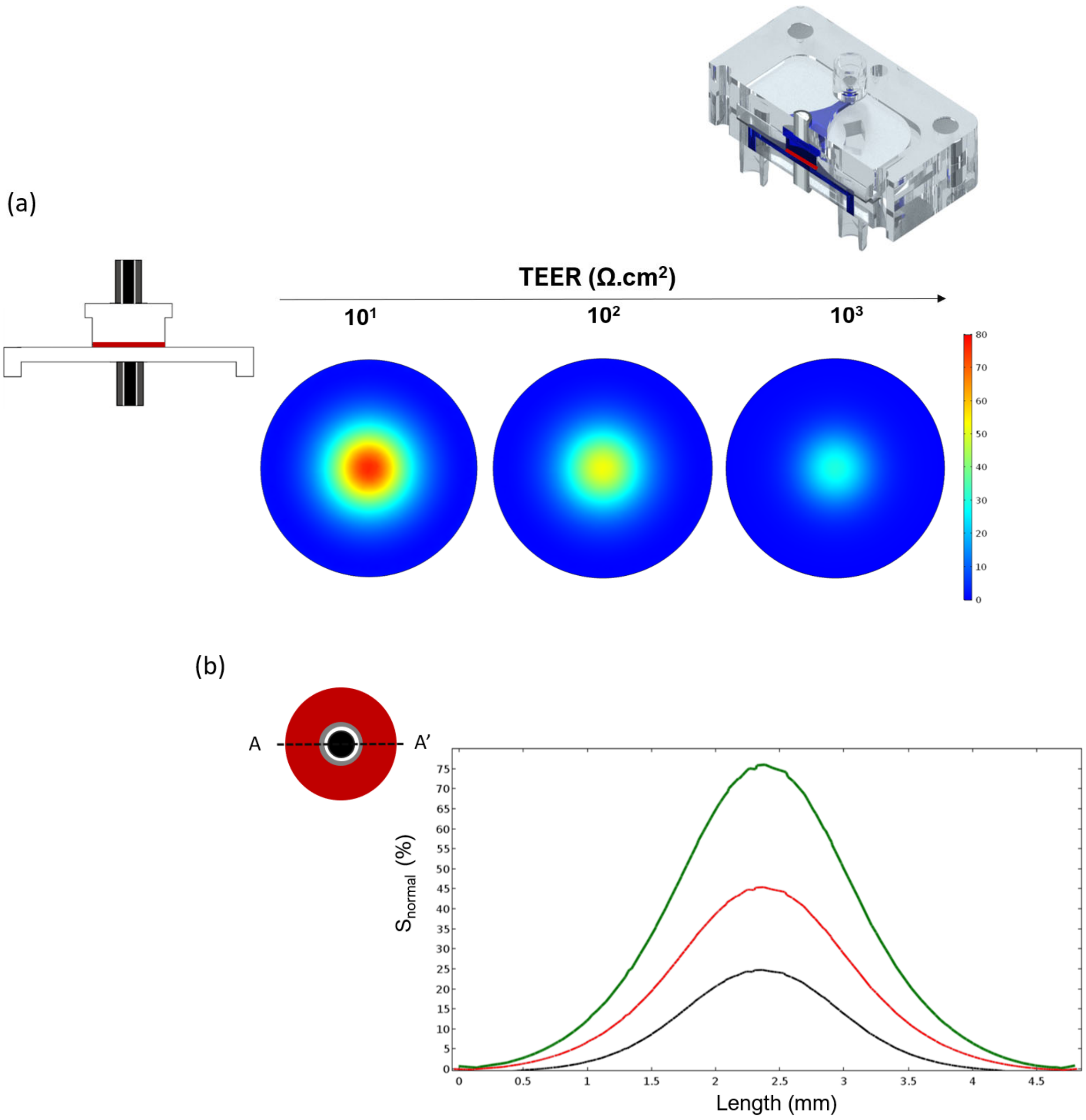

Figure S4. BoC A with asymmetric placement of cell culture insert. Normalized sensitivity distribution (S_{norm}), including the planar sensitivity distribution along the cell barrier surface (rainbow colour map) in the x–y plane and sensitivity distribution along the cell barrier though the axis (lines AA′) shown in the scheme of the model at the left, when TEER is measured. Results are presented for different TEERS (10^1 -10^3 Ω.cm^2). A S_{norm} value of 0% represents the zone of lower sensitivity. Note that axes have different scales.

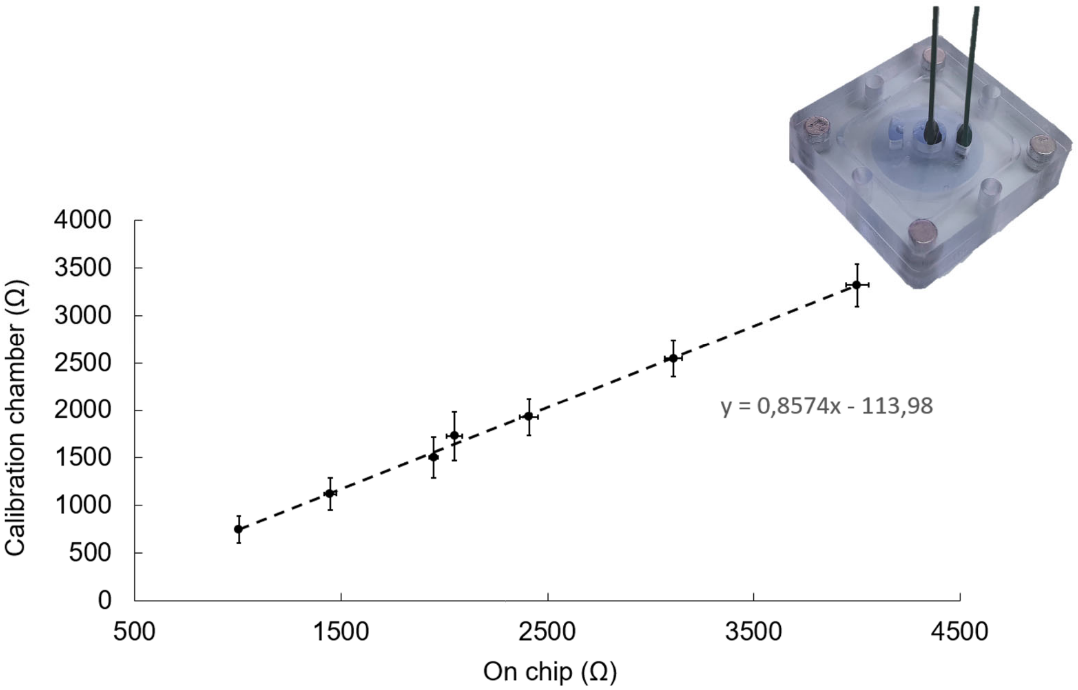

Figure S5. TEER measurement calibration device. Resistance readings from the calibration device and the BoC with integrated electrodes showing a linear correlation. Picture of the assemble device with chopstick electrodes (top right).
